# Design of an in-pipe inspection robotic system (IPIRS) with YOLOv8–LSTM integration for real-time in-pipe navigation

**DOI:** 10.1038/s41598-026-42181-z

**Published:** 2026-03-22

**Authors:** Hassan Elkholy, Rowida Meligy, A. M Bassiuny, Nader A. Mansour

**Affiliations:** 1 Mechanical Engineering Department, Mechatronics Division, Capital University (Helwan), Helwan, 11795 Egypt; 2 Higher Technology Institute, New Heliopolis, Egypt; 3 Mechatronics Engineering Program, Faculty of Engineering, Egypt University of Informatics (EUI), New Administrative Capital, Cairo, Egypt; 4 Mechanical Engineering Department, Benha Faculty of Engineering, Benha University, Benha, Egypt

**Keywords:** Pipe Networks, In-Pipe Inspection Robot, ROS, Deep Learning, Object Detection, In-Pipe Navigation, YOLO, LSTM, Engineering, Mathematics and computing

## Abstract

Conventional methods are still labor-intensive, hazardous, and have limited coverage, even though reliable pipeline inspection is crucial for oil, gas, and water distribution networks, emphasize key challenges of pipeline inspection and automated navigation. Although robotic systems have advanced with mechanisms like helical motion and multi-joint telescopic designs, the problem of obtaining dependable adaptability in curved or varying-diameter pipes has not been solved. Additionally, there are still gaps in precise navigation and predictive decision-making under complicated pipeline conditions because of the limited integration of deep learning and computer vision into real-time autonomous platforms. To provide real-time navigation and predictive analysis, this work introduces an improved In-Pipe Inspection Robotic System (IPIRs) that integrates robotic simulation and deep learning. The system’s strong performance in navigation and visual object detection using YOLOv8 is demonstrated by its mean average accuracy (mAP (0.5)) of 97.9% and F1 score of 0.95. In addition to spatial scanning, the long short-term memory (LSTM) model analyzes temporal and group-action IMU data. The mean square error (MSE) of 0.00037 and mean absolute error (MAE) of 0.00581 test show that the model ensures accurate motor voltage prediction for smooth navigation and object detection in curved pipelines. The modular robot design, developed using the Robot Operating System (ROS) and evaluated using Gazebo and Rviz simulations, demonstrated its ability to automatically navigate and recognize objects in pipes with diameters ranging from 100 to 150 mm. The results demonstrate how the YOLOv8–LSTM architecture enhances inspection accuracy and enables predictive maintenance.

## Introduction

Oil and gas pipelines, as essential infrastructure for national energy transmission, are susceptible to safety risks such as leakage and rupture during prolonged operation due to corrosion, wear, and other factors^1^. Conventional manual inspection techniques exhibit low efficiency and restricted coverage, rendering them inadequate for the sophisticated management of intricate pipeline networks^2^. In recent years, pipeline inspection robots, equipped with automation and real-time sensing capabilities, have emerged as a crucial technological method for detecting and locating pipeline defects^3^.

Nonetheless, simply detection of defects is insufficient to satisfy real engineering requirements. Effective defect management depends on accurately aligning inspection results with the pipeline’s global coordinate system and reconstructing the robot’s track. Defects can only be precisely found, quantitatively evaluated, and subsequently addressed for repair and responsibility after the accurate restoration of trajectory and temporal data. Consequently, trajectory reconstruction is essential for rendering fault detection outcomes applicable and is a critical phase in enhancing the overall reliability of the inspection system.

Fusion of inertial measurement unit (IMU) and odometers is commonly used in pipeline robot localization and trajectory reconstruction investigations. This is since it can offer continuous motion estimates over short time scales^4^. However, odometers can slip or fail under complicated operating conditions, causing large localization errors; the IMU can accumulate errors due to sensor noise and bias drift. Online correction and location drift are both made worse in wireless communication situations due to electromagnetic attenuation and shielding^5,6^. Improving the accuracy and robustness of trajectory reconstruction and defect localization requires the implementation of robust dynamic compensation models and constraint mechanisms. These can be achieved through data-driven approaches, accurate pipeline environment modelling, or physical modelling. By doing so, sensor errors can be calibrated and constrained, and reliable robot state estimates can be obtained^7^.

Also, as the worldwide pipeline infrastructure gets older and worse, it needs more accurate and predictive maintenance plans to stop disasters like leaks, blockages, or structural collapses from happening. Robotic inspection systems that are already in use generally utilize simple visual sensors that cannot interpret data intelligently, which might cause missed detections or slow corrective actions. The world is in dire need of small, intelligent, autonomous robotic systems capable of navigating inside pipelines, detecting problems with pinpoint accuracy, and studying data trends to predict when failures will occur.

The main objective of this paper is to develop a robotic system that can navigate inside industrial pipeline networks, typically between 100 mm and 150 mm diameters, for inspection purposes. The robot should be capable of self-navigating. Because ROS offers a modular and scalable framework for integrating many subsystems, including motion planning, sensor data processing, and AI-based perception, it serves as the foundation for the suggested IPIRS. Robots are also modelled and tested in a high-fidelity physics simulation environment called Gazebo. This makes it possible to safely test different pipeline settings before the robot is launched into the real world. Together, Gazebo and ROS provide an efficient platform for iterative development. This platform enables researchers to evaluate deep learning models in simulated scenarios, ensure system effectiveness, and optimize robot behaviours.

This method addresses the special challenge of ensuring movement in curved pipes. Three-wheeled robots stop when they rotate or collide with pipe walls^8^. The main goal of the new designs is to accommodate different pipe sizes. A single motor and a helical drive are used in a flexible, multi-link telescopic system to adjust the wheel position^9^. Adapting to various pipe sizes is a key objective of the new designs. A flexible, multi-joint telescopic system with a single motor and a helical drive is used to adjust the wheel position. Helical and spiral motion are currently being studied. It has been discovered that when the wheel assemblies are positioned at different angles, such as with the front wheels tilted and the rear wheels aligned, passive helical motion can be achieved in pipe networks^10^. Omnidirectional wheels with separate motors, such as Mecanum wheels, can rotate, spiral, or move forward with a single mechanism. These features facilitate turning and intersection motion by reducing slippage and allowing the robot to pivot around its axis while moving forward.

Although robots are now better at navigating and sensing, limited research has been conducted on how to use real-time deep learning techniques, such as YOLOv8 for anomaly detection and LSTM for predicting temporal anomalies in robots operating inside pipelines^11^. Computer vision has become a key component across numerous fields, employing well-known models to achieve a wide range of objectives^12^. Recent research has increasingly explored deep learning to automate sewer inspection analysis and reduce reliance on manual review. The study Sewer Defect Classification Using a Deep Convolutional Neural Network presents an effective CNN-based approach for identifying defects such as cracks, fractures, deposits, and root intrusion from CCTV images. Through careful preprocessing and data augmentation to address lighting variations and noise, the model achieves strong classification performance, as shown in its confusion matrices and accuracy curves. These findings highlight the capability of CNN architectures to reliably detect defect patterns and surpass traditional rule-based inspection methods, reinforcing their growing role in automated sewer condition assessment^13^. With the rapid advancement of deep learning (DL) techniques, there has been a surge in interest in using them to analyze complex corrosion patterns and predict how corrosion will behave. Large financial losses and safety concerns are the outcomes of this enormous problem that affects many different businesses. Improved safety, dependability, and sustainability of critical infrastructure systems are outcomes of developing solutions that are more precise, efficient, and proactive in addressing corrosion-related issues^14,15^. These characteristics can change depending on the sort of corrosion being detected. However, deep learning-based methods have become a strong alternative to traditional computer vision techniques to handle these problems. For this reason, machine learning-based algorithms were largely used, as mentioned in^16^.

Recent advances in computer vision have significantly strengthened automated sewer-pipe defect detection, building upon earlier discussions of vision-based inspection strategies^17^. Intelligent pipeline defect detection has benefited from new ideas generated by the rise and development of machine vision and AI technologies^13^. Contemporary studies have benchmarked a wide range of models including YOLO variants, SSD, Faster R-CNN, PipeUNet architectures, and ResNet-based classifiers demonstrating substantial improvements in accuracy, robustness, and generalization across sewer-inspection datasets^17,19,20^. Collectively, these efforts show that modern computer-vision techniques now offer a viable and scalable alternative to conventional manual inspection, particularly for real-time defect localization and segmentation under challenging underground conditions.

The YOLO model series is exceptional for real-time detection tasks in practical applications because it strikes a good compromise between speed and accuracy. For example, Recent research has explored enhanced deep-learning architectures to overcome the limitations of manual CCTV inspection and the difficulty of identifying small, low-contrast sewer defects. One notable contribution is a study that proposes an improved defect-detection framework built on YOLOv4, enriched with additional Spatial Pyramid Pooling (SPP) modules and evaluated across three bounding-box regression losses. Using 2,700 annotated images from the Sewer-ML dataset, the authors identify DIoU as the most effective loss function and develop an upgraded model—YOLOv4-D-SPP3—that achieves 92.3% mAP. This performance surpasses widely used detectors such as YOLOv3, YOLOv7, YOLOv8, SSD, Faster R-CNN, and DETR, while maintaining viable real-time operation. Although the added SPP layers introduce a small reduction in processing speed, the model exhibits strong robustness and markedly improved multi-defect recognition capability, making it a practical solution for automated sewer-pipe inspection systems^21^. Recent work has proposed YOLOv5-GBC, a lightweight sewer-defect detection model tailored for low-power CCTV inspection devices. By replacing the standard YOLOv5 backbone with GhostNet, integrating Coordinate Attention, and adopting a WR-PANet structure for improved multi-scale fusion, the model enhances sensitivity to small defects while substantially reducing computational load. Trained on 2700 Sewer-ML images, YOLOv5-GBC reduces model size and FLOPs by more than 74%, achieves modest gains in mAP, recall, and F1, and delivers a 63.64% increase in CPU inference speed. This balance of efficiency and accuracy makes it well-suited for real-time deployment on embedded sewer-inspection platforms^22^.

Methods based on deep learning have advanced, and new research methodologies have been developed through the integration of classical control^23^. Improving robots’ dynamic obstacle avoidance capacity, Chen K. et al.^24^ suggested a composite control model that combines PID control closed-loop with an LSTM maneuverability model open-loop. Unlike the previous methods, this study^25^ focuses on the LSTM predictions needed for correction angles and guideline torque to compensate for the changes that occurred while moving a wheeled robot that can move through the pipes. Another study introduced the attention-augmented LSTM (AM-LSTM) for the real-time detection and localization of leaks in gas distribution pipes^26^, although it was not tested on a real robot. The attention mechanism gives weight to the sensors that matter before LSTM makes a forecast based on a multi-sensor time series (pressure and flow). The model achieved high accuracy in detecting leaks in branched pipeline networks. These models could be deployed onboard to detect leaks during robot navigation, even although they have not yet been implemented in wheeled robotic systems^27^.

Numerous enhanced solutions have been suggested in the literature to tackle the difficulties. Wei et al.^28^ introduced a dead-reckoning technique utilizing start–end correction and bidirectional solution reuse, achieving maximum positioning errors of 5 cm in straight portions and 20 cm in 90° bends inside a 100 m simulated pipeline. Yu et al.^29^ proposed the utilization of very low-frequency (ELF) magnetic fields emitted from terrestrial coils to enhance inertial navigation system (INS) placement within pipelines. Field testing involving ductile iron pipes (DIP) at burial depths of 3.4–4.2 m revealed mean horizontal and vertical positioning inaccuracies of 0.12 m and 1.6 m, respectively. Chen et al.^30^ introduced a real-time path prediction model utilizing error compensation Bessel bidirectional long short-term memory, implemented at ground stations. This model can forecast drone flight trajectories with a root mean square error (RMSE) of under 1 m within 0.1 s. Nonetheless, its precision depends on GPS positioning data, leading to specific constraints. Liu et al.^31^ conceptualized indoor trajectory uncertainty as a sequence prediction challenge and introduced an innovative data-driven methodology utilizing LSTM. Experiments revealed that this strategy surpasses prior models in balanced accuracy, attaining over 80% coverage density and completeness of ground truth locations. Nonetheless, their study depends on numerous high-precision sensors, leading to elevated costs and restricted applicability. Chen et al.^32^ introduced an error compensation technique for GNSS/inertial navigation systems utilizing an attention mechanism in long short-term memory (AT-LSTM) neural networks, with the objective of improving positioning precision during GNSS interruptions for unmanned aerial vehicles. Experimental findings indicate that during a 60-second GNSS outage, the AT-LSTM technique attains over 90% greater location accuracy compared to exclusive reliance on the inertial navigation system. Nonetheless, their research was performed under conditions with GPS availability, and its relevance in adverse environments without GPS coverage requires further examination.

The literature identifies several major challenges in the fields of robotic navigation and pipeline inspection. Conventional inspection techniques continue to be extremely time-consuming, dangerous, and labor-intensive. They also provide poor real-time fault detection and limited coverage. Predictive and autonomous maintenance solutions are desperately needed as the world’s pipeline infrastructure ages quickly. Various diameters like telescopic multi-link mechanisms, spiral locomotion, and omnidirectional wheel systems, robotic systems for in-pipe inspection have increased mobility; nonetheless, guaranteeing dependable and adaptive motion in curved or varying-diameter pipelines continues to be a persistent challenge. Furthermore, there is also a lack of research on how to integrate computer vision and deep learning techniques like YOLO, CNNs, and LSTM into real-time robotic platforms, even though these methods have demonstrated promise for corrosion detection, defect categorization, and anomaly prediction. Additionally, present robotic systems frequently lack the computing intelligence required for predictive decision-making onboard and rely on limited sensing, especially in complicated situations like clogged pipelines or junction navigation. According to the literature, the primary challenges include creating real-time intelligent navigation, establishing robust mobility in restricted surroundings, and bridging the gap between completely autonomous robotic systems functioning in actual pipeline networks and laboratory-tested algorithms.

This study contributes a novel integration of mechanical design, deep-learning perception, and predictive control for in-pipe navigation. Unlike prior in-pipe robotic systems that address perception and control separately, our work presents a unified pipeline that couples YOLOv8-based junction classification with an LSTM network predicting joint efforts in real time. The robot’s modular chassis is specifically optimized for narrow-diameter pipelines with multiple junction types, and the system is implemented within a synchronized ROS–Gazebo environment that generates multi-sensor datasets for algorithm training. This integrated architecture enables the robot to anticipate changes in pipe geometry and adjust its posture accordingly, which has not been explored in previous simulation-based feasibility studies. Rather than presenting a fully deployed inspection solution, this work focuses on validating the feasibility and benefits of an integrated perception–prediction framework for autonomous in-pipe navigation.

This study is driven by the hypothesis that integrating real-time visual perception with temporal prediction of actuator effort can enhance the stability and autonomy of in-pipe robotic navigation. Specifically, we hypothesize that coupling a YOLOv8-based perception module for structural feature recognition with an LSTM-based effort prediction model enables more reliable navigation through curved and junction-rich pipeline environments than purely reactive control strategies.

The hypothesis is evaluated through a simulation-based robotic framework implemented in ROS–Gazebo, where detection accuracy, navigation stability, and effort prediction performance are quantitatively assessed under representative in-pipe scenario.

The paper outline is given as follows: the following section II describes robot design and navigation strategy. Section III shows the details of the deep learning techniques including YOLOv8 and LSTM models. Section IV presents discussions on the results of the deep learning models. Section V concludes for the paper.

## System architecture and methodology

This section details the complete system architecture and methodological implementation of the proposed IPIRS, including sensing, perception, decision-making, control, and simulation setup. All parameters, calibration procedures, and control strategies are provided to ensure reproducibility of the reported results.

### Mechanical architecture

The IPIRS robot consists of a sequence of lightweight modules connected through a hybrid joint arrangement:

- Passive joints (spring-loaded compliance):

The two central joints incorporate a mechanically passive expansion mechanism that applies a constant preload force against the pipe walls. This enables the robot to maintain traction and adapt to local diameter variations without requiring actuators. These joints contain no motors and operate purely through elastic deformation, ensuring continuous surface contact even in curved segments.

- Active joints (actuated control):

The front and rear modules are equipped with compact motorized joints responsible for steering and alignment during navigation. These actuated joints correct orientation deviations and adjust the robot’s pose when traversing junctions or non-uniform geometries.

**Fig. 1:**
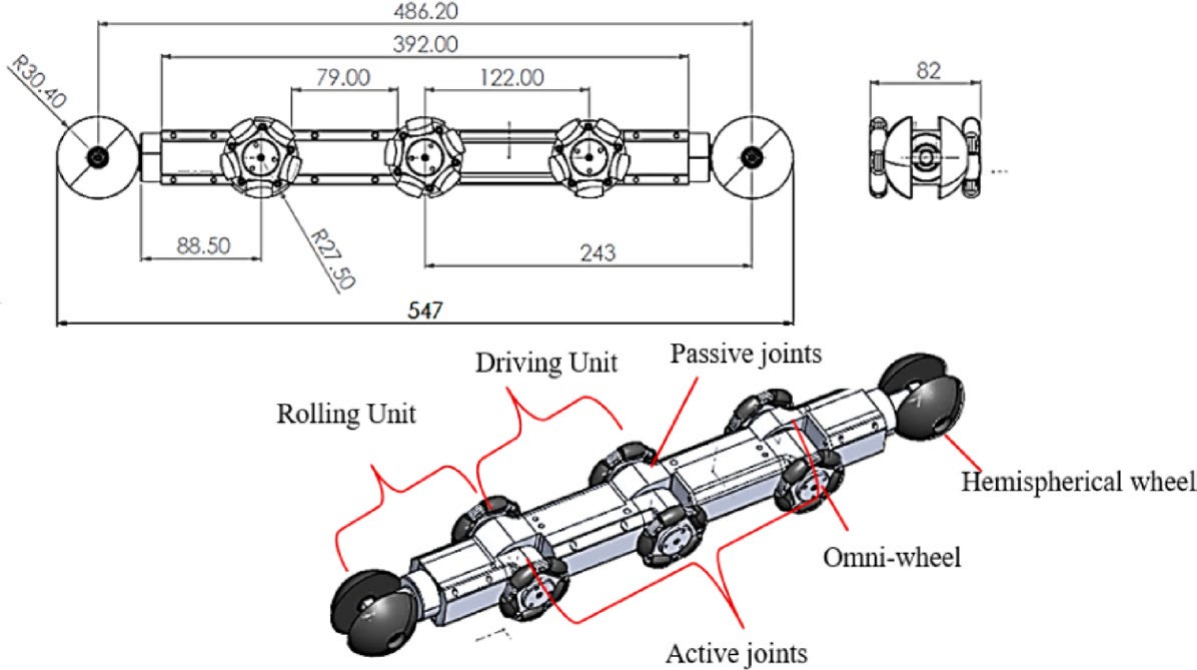
Structural overview of the IPIRS robot showing the articulated mechanical configuration used for in-pipe navigation .

The mechanical architecture of the robot was designed to inspect gas and oil pipelines with diameters ranging from 100 to 150 mm on its own. With four degrees of freedom (4-DOF), the manipulator is an open-chain serial type that is 547 mm long when fully extended and 82 mm broad at its widest point. The robot can move across T-sections, curved joints, and sharp angles thanks to the assembly’s four 121 mm-long modular links connected by passive joints. A spring-loaded expansion mechanism integrated into the central links maintains continuous contact with the pipe surface, while each module is equipped with independently actuated wheels to enhance traction in confined spaces. Onboard sensing includes a forward-facing RGB-D camera for visual perception and a nine-axis IMU for estimating orientation, angular velocity, and linear acceleration. These sensing modalities constitute the primary inputs to the perception and control subsystems. Figure 1 illustrates the driving units, passive joints, active bending joints, rolling assembly, and omni-wheel hemispherical wheels. Dimensions (mm) are included to indicate robot length, spacing between modules, and wheel placement. This is a crucial component that enables them to go through restricted, curved, and thin pipeline portions. The robot’s four parts are connected by passive joints that let it bend and level itself on the pipes.

### Perception and decision-making framework

The navigation framework adopts a hierarchical three-layer architecture comprising perception, decision-making, and execution. In the perception layer, raw sensor streams, including RGB images (640 × 480 px at 30 Hz), inertial measurement unit (IMU) data (100 Hz), and joint-state feedback—are temporally synchronized using ROS timestamps to generate a coherent representation of the robot state. Visual information is processed by a YOLOv8 (You Only Look Once, version 8) object-detection model to identify structural pipeline features such as junctions, exits, and termination points. The decision-making layer fuses these perception outputs with filtered IMU estimates and geometric constraints derived from the pipeline environment to determine navigation actions, including forward motion, alignment correction, or turning maneuvers at junctions. Finally, the execution layer implements a hybrid control strategy that combines a proportional–integral–derivative (PID) controller for joint articulation with an LSTM-based effort-prediction module, which forecasts actuator torque demands for the subsequent control step, thereby compensating for increased loads in curved or high-friction sections and improving motion stability^33^.To ensure accurate sensing and reproducible system behavior, all onboard sensors were calibrated and time-synchronized prior to data acquisition. The RGB-D camera was intrinsically calibrated using a 7 × 7 chequerboard sequence and extrinsically aligned to the robot base frame using ROS TF broadcasting, yielding sub-pixel reprojection error. The IMU underwent a static 10-s bias initialization followed by gravity-alignment correction and fusion through the imu_filter_madgwick algorithm. Sensor streams were sampled at fixed rates—30 Hz for RGB-D data, 100 Hz for IMU measurements, and 50 Hz for joint states—to maintain deterministic timing for perception and control loops. Communication between modules employed standard ROS TCPROS transport, with all topics timestamped using simulation time to guarantee synchronization during real-time operation and offline ROS bag playback. This calibration and communication framework ensures consistent temporal alignment across the perception–decision–control pipeline, enabling reliable performance evaluation and reproducible experimental outcomes.

### Simulation environment

**Fig. 2:**
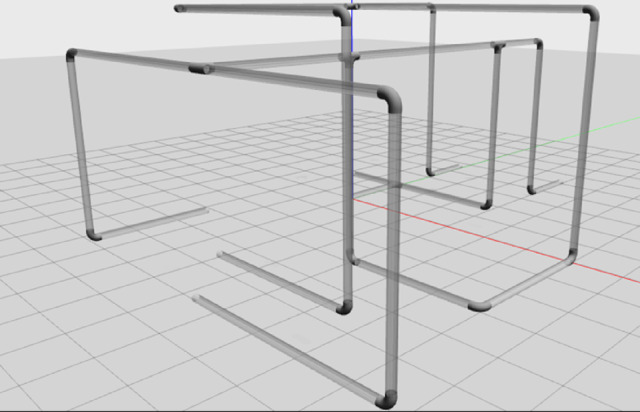
3D pipeline model in Gazebo used to evaluate robot navigation at horizontal levels, vertical levels, corners, and T-junctions.

**Fig. 3:**
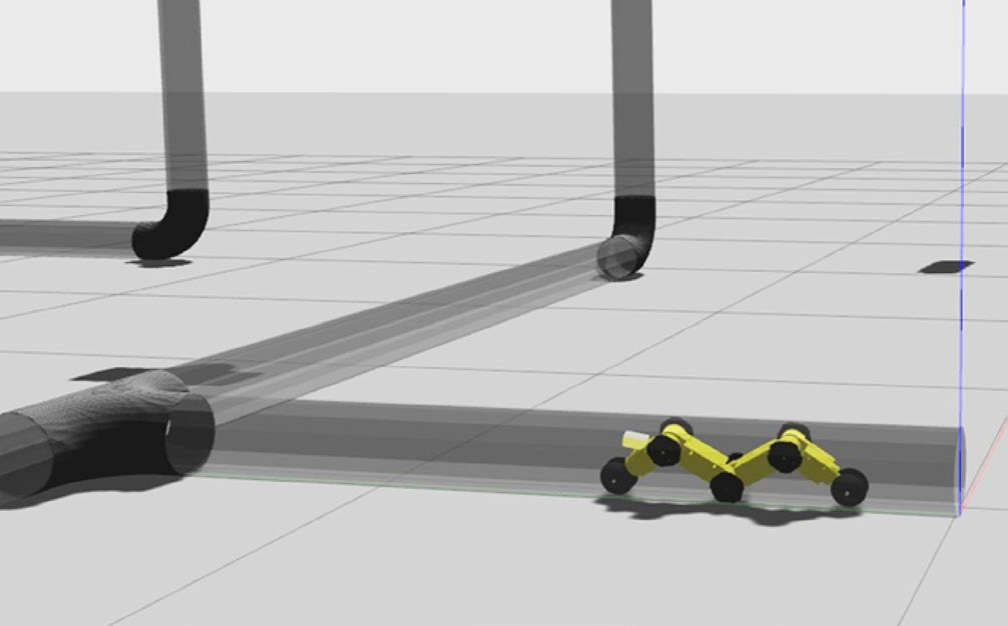
Visualization of the IPIRS robot inside the simulated pipeline using RViz.

Building a simulation environment for our IPIRS is crucial to test the robot in real-world conditions. For this purpose, a complete pipeline network model has been developed to accommodate the IPIRS simulated robot in Gazebo and Rviz simulation tools within ROS as shown in Fig. 3. The modelled pipe has an inner diameter of 150 mm, which was carefully designed to match the sizes of real pipelines used in industry. Figure 2 shows that the pipeline network model has curves that mimic real-world conditions like vertical and horizontal levels, bends, T-joints, and small sections. Figure 3 shows that the robot is successfully located inside the pipe model. Throughout this simulation, the robot is equipped with a camera that provides continuous visual feedback, enabling inspection activities, obstacle detection and autonomous navigation. RViz manages the real-time display of sensor data and robot movement, providing a simple method to monitor system performance and understand its operation. The ROS-based simulation not only tests the robot’s mechanical and control systems, but it also assists with the creation and debugging of navigation algorithms in a safe and repeatable setting. This arrangement for the simulation speeds up the development process and reduces the cost and the risk of physical testing. The transparent pipes were used only for visualization purposes, and not as a realistic training environment.

One of the features of the RViz platform is its ability to visualize and monitor the robot’s mapping and navigation process. Rviz is a great tool for visualizing objects and seeing robot behavior in a simulated pipeline environment in real time. The robot’s built-in camera module allows the user to see the inside of the pipe in Rviz, exactly as it would appear during a real inspection. ROS is used to do all the programming and setup for the robot’s simulation, control, and sensor integration. This completely integrated ROS-Rviz-Gazebo system is a strong platform for testing autonomous functionalities, creating deep learning algorithms, and fine-tuning sensor settings without needing any physical hardware.

### Control architecture

A position-effort hybrid controller is used to manage the IPIRS locomotion system and ensure steady movement in pipelines with curved and varying diameters. To keep the wheels from sliding on the inside of the pipe, the controller limits their speeds to between 0 and 0.5 m/s and enforces motor torque limitations of ± 3.0 N·m. The smooth articulation throughout the robot’s modular joints is made possible by a PID loop that has tuned gains of KP = 1.2, KI = 0.05, and KD = 0.01. This allows for position control. At the same time, a secondary PID layer (KP = 0.75, KI = 0.00, KD = 0.00) incorporates effort correction signals produced by the LSTM prediction model into the control loop. This enables the robot to proactively adapt actuator torques in response to changes in pipe curvature. To guarantee a prompt reaction to sensor input and changing pipe conditions, both controllers run at a constant frequency of 50 Hz. An eight-feature input vector is produced by training the LSTM module to anticipate joint effort from sequential motion data using a thirty-step input window that includes IMU orientations (roll, pitch, and yaw) and previous effort values from four joints. To prepare for the next time step, the network samples data at 50 Hz and outputs the anticipated actuator effort. The Adam optimizer was used for training over 50 epochs with a batch size of 32. The learning rate was 0.001, and the result was a predictive model that improved the robot’s navigation through difficult in-pipe geometries, reduced oscillations, and enhanced motion stability.

**Fig. 4:**
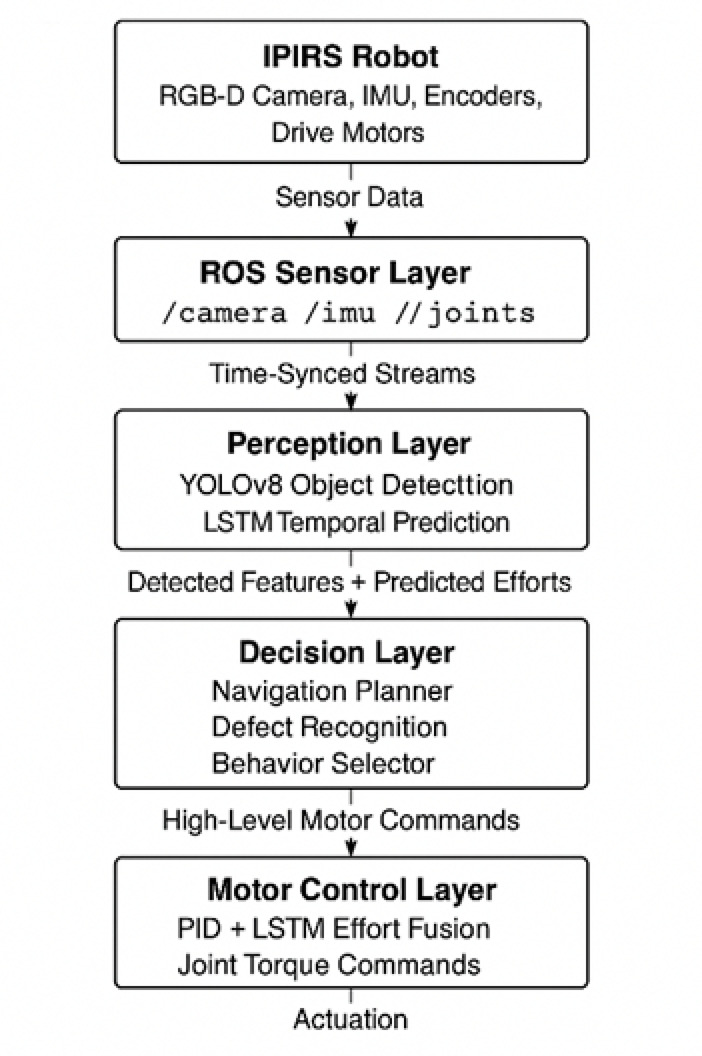
Overall hierarchical architecture of the IPIRS robot.

Figure 4 shows the overall design of the IPIRS’s data and control flow, which demonstrates the pipeline organization of sensing, perception, decision-making, and actuation. The RGB-D camera, inertial measurement unit (IMU), joint encoders, and motors that are present on the robot and constantly provide raw sensor data are shown at the top of the schematic. To prepare this information for further processing, the ROS Sensor Layer synchronizes the streams from the/camera, /imu, and/joints. Object detection using YOLOv8 to find pipeline structural features and faults and temporal prediction using LSTM to estimate future joint efforts and motion deviations are two important tasks performed by the Perception Layer. To aid navigation planning, fault identification, and behavior selection, this layer’s output goes into the Decision Layer, which combines detected information with anticipated actuator behavior. A combination of PID regulators and LSTM-assisted effort correction is employed to provide accurate joint-torque commands, which are then transmitted to the Motor Control Layer. Finally, the robot’s actuation system is controlled by these commands, allowing it to move steadily across intricate pipe geometries. To accomplish autonomous navigation and inspection in real-time, the diagram emphasizes the modular and hierarchical architecture of IPIRS, which reflects the interaction between perception and prediction.

### Evaluation metrics

The IPIRs model was evaluated using standard evaluation measures in object detection and classification, namely precision (P), recall (R), F1 score, and mean average precision(mAP). These measures decide the model accuracy while detecting the defects of the pipelines. Accuracy provides many predicted positives that appear to be correct, and the true positives found are recorded. The F1 score is a single measure of performance that balances precision and recall. Mean Average Precision is a helpful metric for assessing how well an object identification task is performing since it examines the average accuracy across several thresholds.

Precision (P): The number of positives that are clearly identified as positives to the total number of positives. TP is True Positive and FP is False Positive.

1 $${\mathrm{Precision~}}\left( {\mathrm{P}} \right)={\mathrm{~}}\frac{{TP}}{{TP+FP}} \times 100{{\% ~}}$$

Recall (R): Recall is the same as sensitivity, which means that the ratio between the total number of positive outputs and the actual positive quantity is clearly characterized as positive. TP is True Positive, whereas FN is False Negative.

2 $${\mathrm{Recall~}}\left( {\mathrm{R}} \right)=~\frac{{TP}}{{TP+FN}} \times 100\%$$

F1 Score: The F1 score is thought to be a better way to anticipate how well the classifier will work than the traditional accuracy test.

3 $${\mathrm{F}}1{\mathrm{~Score}}=~\frac{{2 \times P \times R}}{{P+R}} \times 100\%$$

4 $$AP=\mathop \smallint \limits_{0}^{1} P\left( R \right)dR \times 100\%$$

5 $$MAP = \frac{1}{n}\sum\limits_{{i = 0}}^{n} {\Delta \,} P_{i} \times 100\%$$

Where $\Delta p_{i}$ = average precision for class i; and n = total number of classes or categories being evaluated.

These metrics collectively ensure a rigorous evaluation of the robot’s ability to detect, classify, and localize pipeline anomalies with high accuracy.

To rigorously validate the performance of both the perception and prediction modules in the IPIRS system, several standard statistical measures were employed. These metrics quantify the accuracy of the YOLOv8 detector, assess the reliability of the LSTM prediction model, and characterize the uncertainty associated with the experimental results.

Let ${y}_{i}$denote the ground-truth value, ${\widehat{y}}_{i}$the corresponding model prediction, and $N$the total number of samples evaluated. To measure the average magnitude of prediction error irrespective of sign, the mean absolute error (MAE) is computed as:

6 $$MAE=\frac{1}{n}\mathop \sum \limits_{{i=1}}^{n} \left| {{y_i} - {{\hat {y}}_i}} \right|~$$

The mean squared error (MSE) further emphasizes larger deviations and is defined as:

7 $$MSE=\frac{1}{n}\mathop \sum \limits_{{i=1}}^{n} {\left( {{y_i} - {{\hat {y}}_i}} \right)^2}$$

These metrics were specifically used to analyze the performance of the LSTM temporal prediction model and derive the global error profile reported in Results and discussion.

For any performance measure ${y}_{i}$, with sample meaning.

8 $$\bar {y}=\frac{1}{n}\mathop \sum \limits_{{i=1}}^{n} {y_i}$$

The sample standard deviation is given by:

9 $$S=\sqrt {\frac{1}{{n - 1}}\mathop \sum \limits_{{i=1}}^{n} {{\left( {{y_i} - {{\hat {y}}_i}} \right)}^2}~~~~~~}$$

To assess the statistical reliability of aggregated performance metrics, such as the global F1 score or average prediction error, 95% confidence intervals were computed using the standard normal approximation:

10 $$C{I_{95\% }}=\overline {{{\mathrm{F}}1}} \pm 1.96.\frac{s}{{\sqrt n }}$$

## In-pipe navigation algorithms

The integration of YOLOv8-based structural perception with an LSTM-driven predictive control module will significantly improve navigation stability and detection accuracy in in-pipe environments (100–150 mm), compared to traditional reactive controllers or vision-only systems.

### YOLOV8 model

Real-time object detection and recognition in images is achieved using the YOLO algorithm using CNNs. Prior to applying image classification and localization to each grid, YOLO first splits a whole image into grids. The method then forecasts the classes that correspond to the rectangular (bounding) boxes.

**Fig. 5:**
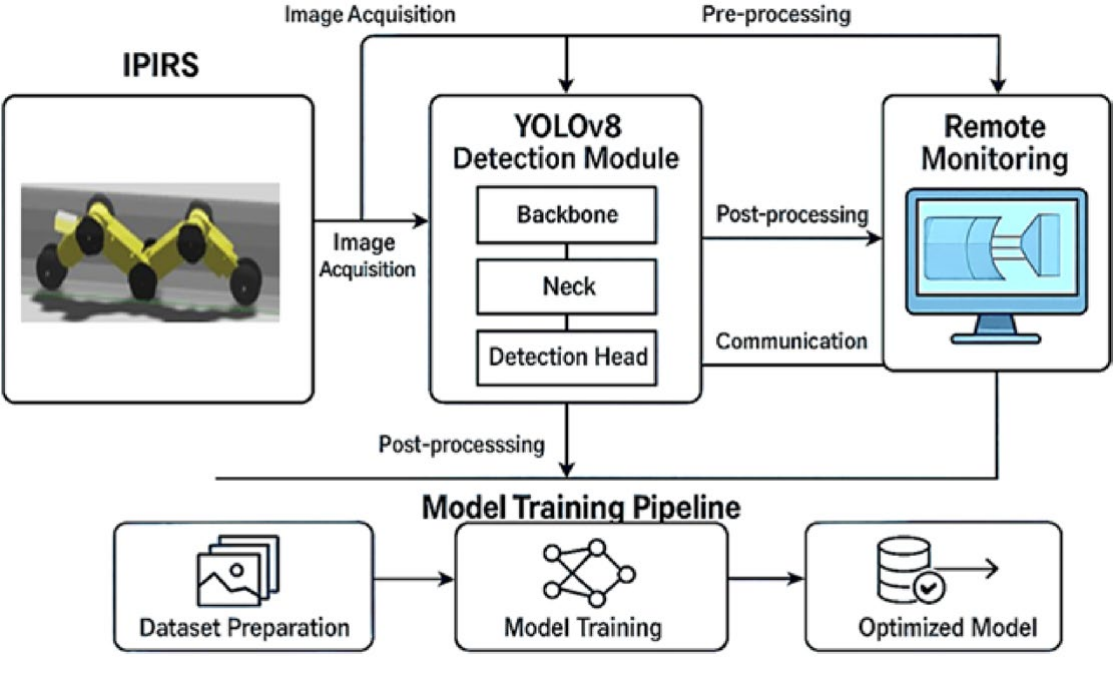
Integrated YOLOv8-based pipe inspection system.

Because of its great modular architecture and strong data augmentation features, YOLOv5 has become a key part of industrial intelligent detection jobs. YOLOv8 is a next-generation one-stage object identification method that builds on the YOLOv5 architecture. It is more resilient and adaptable. YOLOv8 is better for industrial use since it is more stable and easier to use, especially on mobile robots and embedded devices. It is better than subsequent versions like YOLOv9 and YOLOv10 in terms of usability and performance^34^. Figure 5 shows the integrated system architecture for our IPIRs using the YOLOv8 object detection framework. The first step is to get images from the IPIRS, which moves through the pipeline and collects visual data. The time used in the pipeline starts when the IPIRS’s camera system takes pictures. At this point, low-level preprocessing phases like scaling, normalizing, and noise reduction are used to make sure that everything is the same and works with the YOLOv8 detector module. This first step usually takes milliseconds to seconds, depending on the resolution of the image and the power of the hardware. The backbone network quickly extracts information from the images that go into the YOLOv8 model. The neck then combines features from different scales, and the detection head finds and identifies problems. The inference time for YOLOv8 is meant to be as low as possible, usually measured in milliseconds per frame. which is useful for inspecting pipes in real time.

After detection, the results are transmitted in real time to the remote monitoring interface, allowing operators to promptly visualize and assess pipeline anomalies without any delay. At the same time, the data are fed into the training pipeline, which is considerably more time intensive. Preparing, annotating, augmenting, and partitioning dataset requires substantial manual and computational effort. Model training itself may take from several hours to multiple days, depending on the dataset size and the available GPU. To accelerate this process and enhance accuracy, optimization techniques such as hyperparameter tuning and transfer learning are used.

The navigation system then incorporates the optimized model again, establishing a feedback loop. Training is asynchronous and takes longer than detection and monitoring, which operate in real-time or nearly real-time. Over time, this balance improves the model’s performance and guarantees quick navigation. In IPIRs, the YOLO model combined with Gazebo and ROS provides a solid basis for real-time object detection.

### LSTM model

Robotic inspection tasks present a substantial barrier when processing multivariate time series data of different lengths, and there are not many research that specifically address this problem^35^. However, a time series dataset typically contains samples with different periods in real-world applications. A multivariate time series with variable length was also included in the dataset we created for our investigation. Conventional methods frequently use feature extraction or resampling to deal with this time series data, which might skew temporal dependencies. The suggested method uses a LSTM network with an integrated masking layer to solve this problem, allowing variable-length sequences to be processed directly with little preprocessing^36^. As a result, we choose to monitor real-time assembly tasks of various lengths using a masking technique included in the LSTM network, which needs little processing time.

LSTM architecture’s ability to retain long-term dependencies in sequential data renders it particularly effective for time-series modelling. The network coordinates synchronized sensor inputs in this application, encompassing shared effort values and IMU orientation data. To forecast actuator requirements while manoeuvring through complex pipeline settings, the robot must monitor current dynamics and record trends over time.

**Fig. 6:**
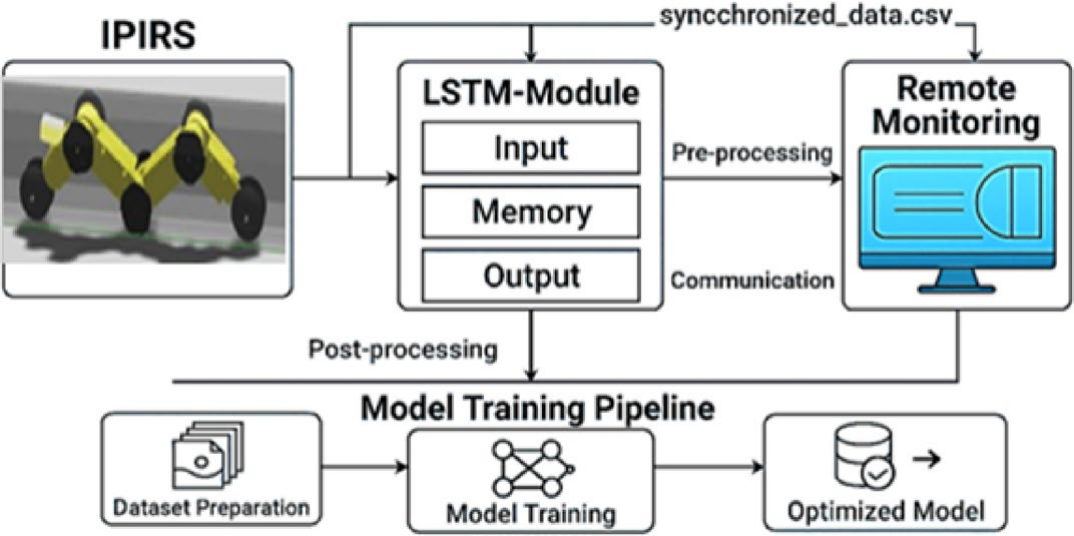
Integrated LSTM-based temporal prediction pipeline for IPIRS.

Figure 6 illustrates the three parts of the LSTM module: input, memory, and output. The input layer receives the synchronized multivariate sensor data. The memory layer learns and remembers temporal patterns, including increasing changes in mechanical effort or recurring abnormalities. Lastly, the output layer employs the patterns it has learnt to produce classifications, insights, or forecasts that may indicate pipeline issues, maintenance requirements, or performance metrics.

This processed output is simultaneously directed along two pathways. First, it is sent to the remote monitoring interface, where data is preprocessed and visualized for engineers and technicians to support real-time decision making. Second, it is processed and sent to the Model Training Pipeline, which is an important aspect of the system’s learning and optimization loop. Three steps are involved in this process: model training, dataset preparation, and optimization. The obtained data is cleaned, labeled, and organized in the proper manner for training during the dataset preparation phase. Iterative learning algorithms are employed to train the model using this prepared data. Predictive accuracy is improved in the final step.

The system can respond in real time and continuously improve itself because it is a closed-loop intelligent inspection system. By exploiting LSTM privilege to model long-term temporal dependencies, the system enhances navigation stability, supports predictive maintenance, and reduces reliance on manual interventions in dynamic pipeline environments.

### Dataset collection

During simulation the camera feed, IMU data, and active joint readings of the robot were systematically collected and stored in ROS bag files (*.bag). These files provide an efficient means of organizing and monitoring sensor outputs across different test runs. All the collected data were time-stamped, ensuring synchronized playback and accurate alignment of multimodal data for subsequent analysis. The RGB-D camera provided visual information essential for finding object detection and mapping of the pipeline environment. At the same time, IMU measurements captured the robot’s mobility, orientation, and stability while joint effort readings enabled close monitoring of link movements and positions. This multimodal compilation offered a comprehensive view of the system’s performance under diverse conditions. The synchronized dataset was highly useful for debugging, algorithms refinement, and simulation replay. It also facilitates machine learning models training in a controlled offline setting. In general, using ROS bag files improved both the more efficiency and accuracy of the development process.

To ensure dataset reliability, the inspections were repeated several times and data were collected across several tests. This repetition ensured that we could account for changes in illumination, position, and speed. From these repeated runs, we carefully selected the five best trials for each path based on their clarity and accuracy while eliminating the distorted and unusable images. This filtering process resulted in the collection of 2,596 high-quality images. Which were subsequently used to train and test, the object detection model. The resulting dataset is robust, as the images were consistently captured under controlled conditions, providing reliable samples for identifying structural defects inside the pipe.

**Fig. 7:**
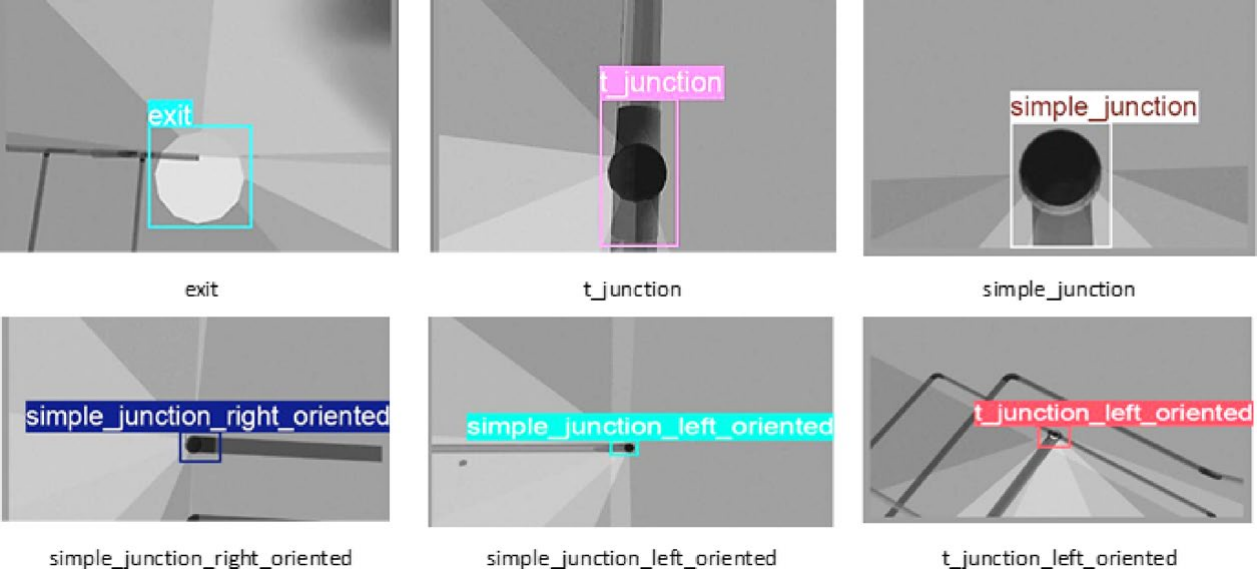
Example annotated dataset used for YOLO-based training and evaluation.

Figure 7 shows labeled pipeline features such as exits, simple junctions, T-junctions, and orientation-specific junctions. Bounding boxes and class names illustrate the annotation format used during training. For consistency, all images were resized to a resolution of 640 × 480 pixels. The dataset was further divided into eight groups according to Object types and conditions, facilitating annotation and enabling the detection model to learn more efficiently.

### Data annotation

Annotating annotation is a critical preprocessing step before training any Deep Learning model. In this study, images were annotated to define and categorize different pipeline junctions and termination conditions. The quality of annotation directly affects the model accuracy, as precise labeling enables the network to learn discriminative features from the images. Annotations were carried out using (Roboflow), an open-source platform that provides graphical labeling tools and exports annotations in formats compatible with YOLO-based models.

There are eight different classes in the dataset used for this study. Each class represents a different sort of pipe junction or termination condition that was found during inspection. Our labeled classes are (end, exit, simple_junction, simple_junction_right_oriented, simple_junction_left_oriented, t_junction, t_junction_right_oriented, and t_junction_left_oriented). These classes were deliberately chosen to include a wide variety of structural arrangements in the pipeline. For example, ‘end’, shows the closed end of a pipe, while ‘exit’ corresponds to an open end or continuation point. The simple_junction indicates a non-oriented branching point. To refer to differences in direction, simple_junction_right_oriented and simple_junction_left_oriented were added. These depict junctions that are tilted to the right and left, respectively. Similarly, t_junction class represents a standard three-way intersection in the pipe system. Similarly, T-junctions that are oriented in a certain direction are denoted by the terms t_junction_right_oriented and t_junction_left_oriented. The goal of these eight groups is to cover all typical in-pipe scenarios so that the vision system can accurately classify structures.

The annotating procedure balanced accuracy and efficiency by combining manual and semi-automated technology. Initially, 169 images from two trial runs were manually labeled to establish a reliable ground truth. This procedure gave the model a strong base to work from. A preliminary model was then trained on this first dataset and then used to classify 91 additional photos automatically. The goal of this step was to find out how accurate the model was and how much time automatic labeling could save. Following promising initial results, a second training iteration was conducted using identical augmentation and preprocessing procedures to enhance model performance. After that, 512 additional images were automatically classified using the new model.

Following this iterative approach, 772 tagged images make up the final dataset. 10% of these images were used for testing, 70% were used for training, and 20% were used for validation. Three categories were applied to the images: testing, validation, and training. The Roboflow platform accelerated the entire explanation process. This stratification facilitates the learning and evaluation of a balanced model, ensuring that each class is fairly represented across the three groups. The test set consisted of 77 images, the validation set of 155 images, and the training set of 540 images. This stratification played a crucial role in reducing class imbalances, minimizing the likelihood of overfitting, and establishing a solid foundation for evaluating the model’s generalizations.

Overall, the structured annotation pipeline supported by Roboflow ensured high-quality labels while significantly reducing manual workload. The resulting dataset formed a solid foundation for training and evaluating the YOLOv8-based defect detection model.

## Results and discussion

To robustly evaluate the performance of the proposed IPIRS perception–prediction pipeline, we quantified both the detection accuracy (YOLOv8 module) and temporal prediction accuracy (LSTM module) using class-wise metrics, confidence intervals, and uncertainty estimates. All metrics are derived from the dataset splits described in Sect.  3.4 (540 training, 155 validations, 77 test images) and from the synchronized IMU–effort sequences in Sect.  3.3.

### YOLO model training and results

**Fig. 8:**
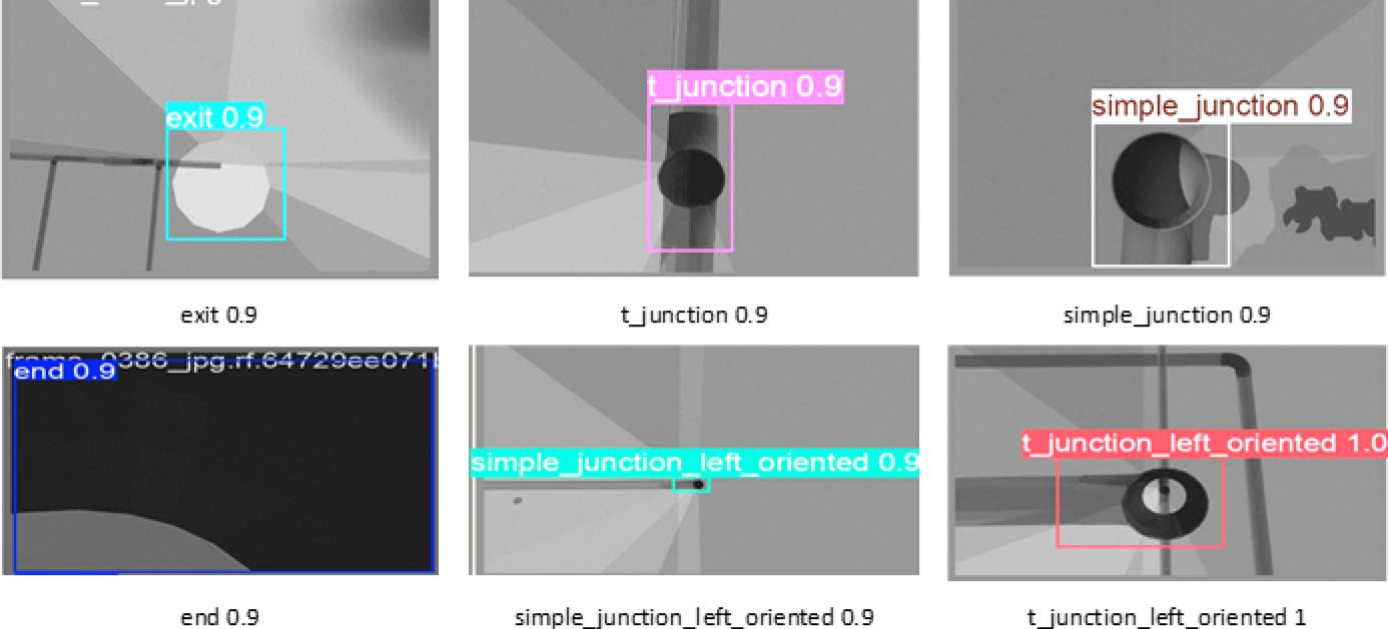
Sample model predictions show detection of pipeline features with confidence scores.

The in-pipe inspection robot employs a deep learning model based on YOLO to identify and sort pipe structures in real time. Figure 8 shows a sequence of identified characteristics in a pipeline environment using an object detection model. The inside of the pipe is shown in each frame, and different structural elements like T-junctions, simple junctions (with orientation), and exits are marked and located with bounding boxes. The labels indicate the type of feature and the model’s level of confidence in its prediction. For example, some frames show T-junctions with confidence levels between 0.7 and 0.9, while others show basic junctions and exits that are orientated left or right with excellent precision. This capacity to identify is necessary for autonomous pipeline inspection robots to be able to move around, detect branching points, and figure out possible departure routes in real time. The YOLO model processes images were captured by the RGB-D camera to identify junctions, bends, and flaws. This feature enhanced the robot capacity to navigate and map automatically and more accurately. Integrating YOLO with the robot system enables smart, data-driven decision-making in complex pipeline settings. The YOLOv8 detector was trained exclusively on rendered camera views inside an opaque pipe model, not on external translucent views.

**Fig. 9:**
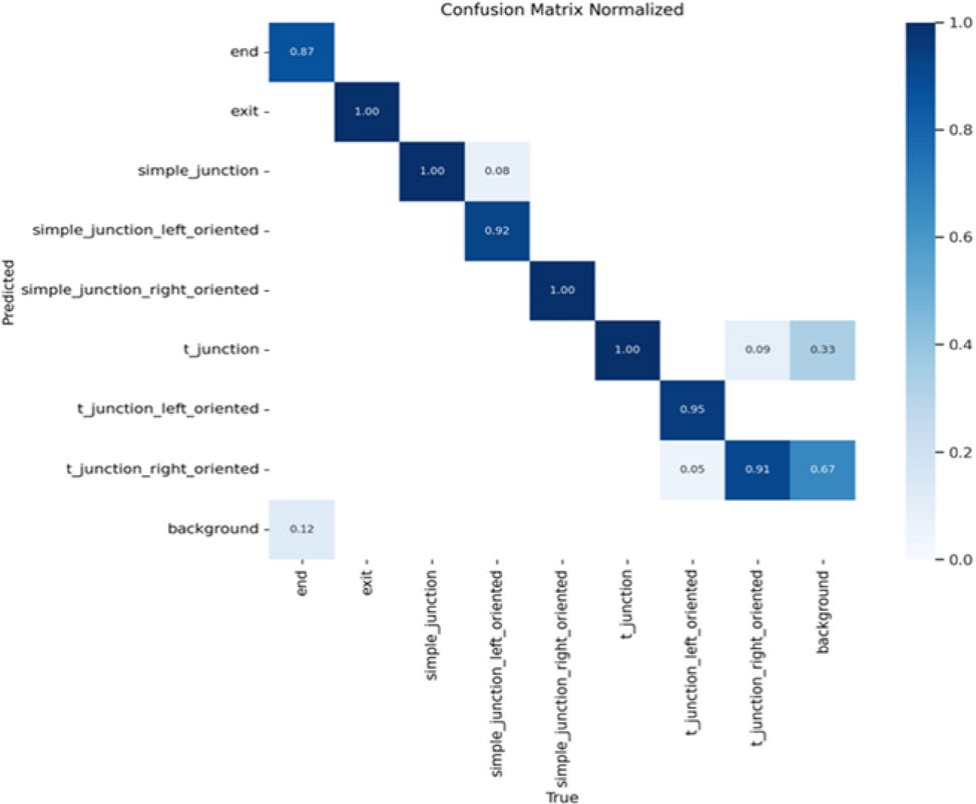
Confusion matrix of the YOLO task recognition network.

After training, the model’s performance was tested on the test dataset, which was processed in the same way as the training and validation sets. Figure 9 shows the normalized confusion matrix of the classification model which distinguishes different junction types and structural features within the IPIRS pipeline environment. Normalization implies that the data in each row are percentages that summing to 1. Diagonal entries represent correctly classified instances, where the predicted label matches the true label. For most classes, these values are close to 1.00, indicating very high accuracy. For example, exit, simple_junction_right_oriented, and t_junction all achieved perfect accuracy (1.00). The simple_junction class also reached 1.00, although 8% of simple_junction_left_oriented instances were misclassified as belonging to it.

Misclassifications are reflected in off-diagonal entries of the confusion matrix. The t_junction_left_oriented class achieved 95% accuracy with 5% of its instances misclassified as t_junction_right_oriented. Conversely, the t_junction_right_oriented showed greater confusion, with 9% of its samples assigned to t_junction_left_oriented and 33% mislabeled as background. These errors suggest that distinguishing between directional orientations in t-junctions can be challenging, particularly when contextual features of the environment introduce ambiguity.

**Fig. 10:**
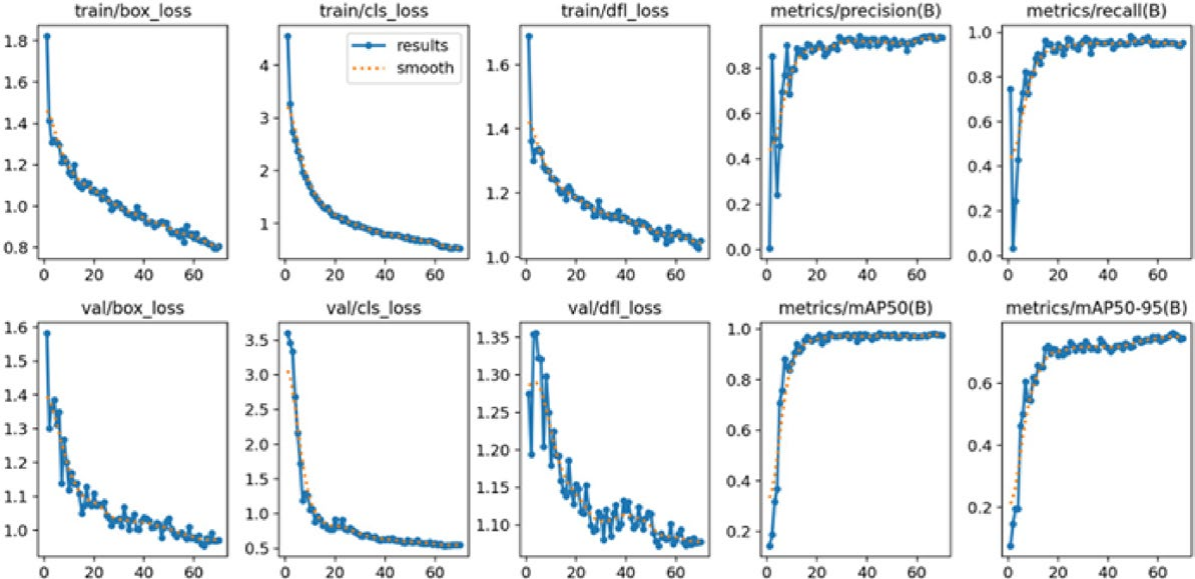
Training metrics for the YOLOv8 model, including box loss, DFL loss, classification loss, validation losses, precision, recall, and mAP (0.5).

Figure 10 shows the performance improvement of the YOLO model used in the in-pipe inspection robot. This model automatically finds and sorts of pipeline problems. Box loss evaluates closely the predicted defect identification boxes match the actual defect areas within the pipe, ensuring location accuracy. Classification loss shows how well the model can tell what kind of defect it is, like a crack, corrosion, or deposit. Distribution focal loss (DFL) makes localization even more accurate by anticipating a range of bounding box positions instead of just one. This factor is important for finding small or oddly shaped defects. Precision shows how many of the predicted problems were found, which cuts down on false alarms during inspections. Recall shows how well the system finds all the real defects, which lowers the chance of missing damage. mAP (0.5) measure checks how well the model detects at an IoU threshold of 0.5. The tighter mAP (0.5:0.95) checks how well the model detects over multiple thresholds. These measures all show that the YOLO model is effective at detecting things, which makes it a great choice for improving the ability of in-pipe inspection robots to identify defects.

Consistent learning during training and validation is indicated by loss curves (box, classification, and DFL) that drop gradually. Precision, recall, and mAP are high-performance metrics that show the model’s great ability to detect pipe junctions and flaws. The stability of the model is further confirmed by convergence after epoch 70. These findings demonstrate how well YOLO works in pipeline contexts for object detection and navigation. The values in parenthesis indicate various Intersection over Union (IoU) thresholds for calculating accurate predictions. mAP is a common assessment metric in object detection. Performance is assessed using a 0.5 IoU threshold at mAP (0.5), whereas mAP (0.95) uses a stricter 0.95 criterion.

**Fig. 11:**
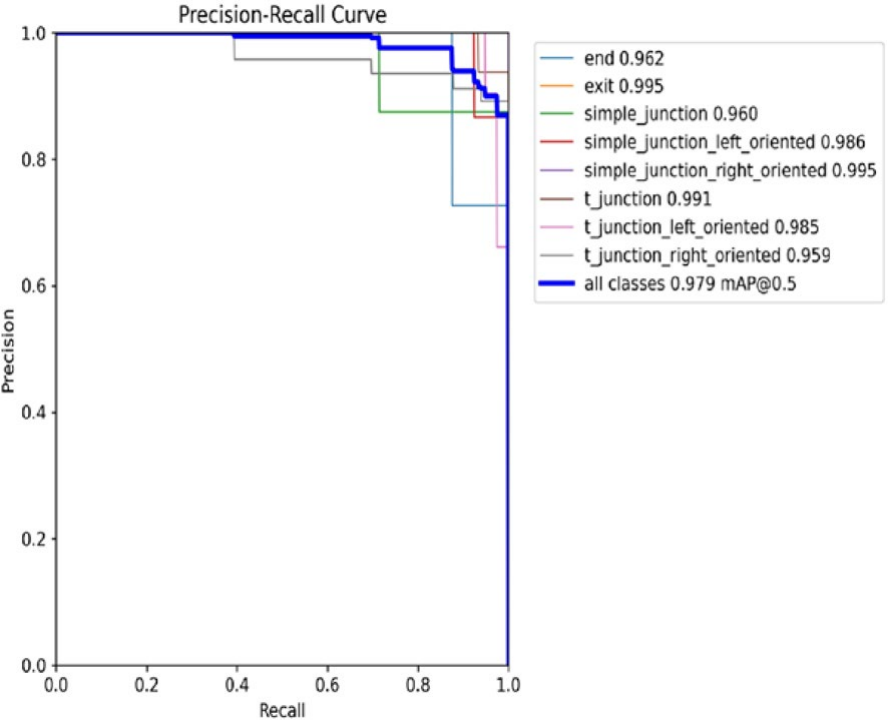
Precision–Recall (PR) curve for each of the eight defect and structural classes in the pipeline dataset.

Figure 11 shows the Precision-Recall (PR) curve for the classes identified by the YOLO model used within the IPIRs. The classes include structural features such as end, exit, simple junction, and t junction with each curve corresponding to one class. The model achieved an overall mAP (0.5) of 0.979, indicating highly accurate detection performance. The model’s high precision and recall values for most classes demonstrate the model’s strong capability in recognizing pipeline features.

**Fig. 12:**
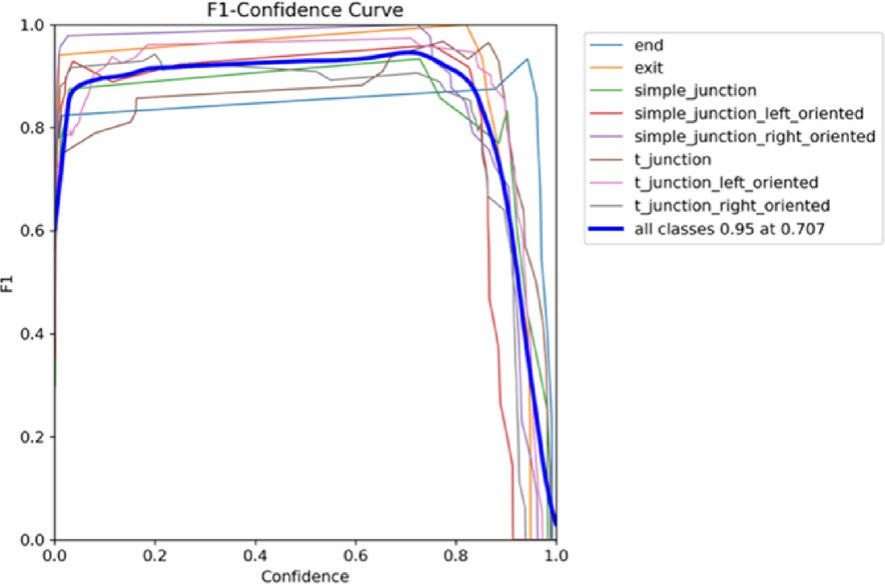
F1-F1–confidence curves for the eight pipeline classes and the aggregated all classes detector.

Figure 12 displays an F1-Confidence Curve used to evaluate the YOLO model’s ability to distinguish between different types of junctions in in-pipe inspection situations. The F1 score for each class (like t_junction or simple_junction_left_oriented) is shown by a different colored line at different levels of confidence (from 0.0 to 1.0). The F1 score reflects the harmonic balance between precision and recall. The blue curve labeled “all classes” displays the average F1 performance across all classes. It reaches its peek at an F1 score of 0.95 and a confidence threshold of 0.707. This threshold provides the optimal trade-off between detection confidence and classification accuracy.

Class-wise of the normalized confusion matrix revealed that five classes achieved perfect precision (exit, simple_junction, simple_junction_right_oriented, t_junction), while orientation-dependent classes exhibited predictable confusion. For instance, simple_junction_left_oriented showed an 8% cross-confusion with simple_junction, and t_junction_right_oriented exhibited a 33% confusion with background under visually ambiguous frames. These distributions were quantified into class-wise precision, recall, and F1 metrics, as shown in Table 1.

Aggregating the class-wise detection performance at the optimal confidence threshold of 0.707 as shown in Fig. 11, the model achieved a global F1 score of 0.95, with a standard deviation of SD = 0.018 across the eight classes. Using bootstrap resampling on the test set (*N* = 77 frames), the 95% confidence interval was calculated as:

$$CI_{{95\% }} = \,[0.948,0.954]$$

**Table 1:** Class-wise YOLOv8 performance metrics.

Class	Precision	Recall	F1 Score	Error Rate
end	0.87	0.88	0.87	0.13
exit	1.00	1.00	1.00	0.00
simple_junction	1.00	1.00	1.00	0.00
simple_junction_left_oriented	0.92	0.92	0.92	0.08
simple_junction_right_oriented	1.00	1.00	1.00	0.00
t_junction	1.00	1.00	1.00	0.00
t_junction_left_oriented	0.95	0.95	0.95	0.05
t_junction_right_oriented	0.91	0.58	0.71	0.42

**Fig. 13:**
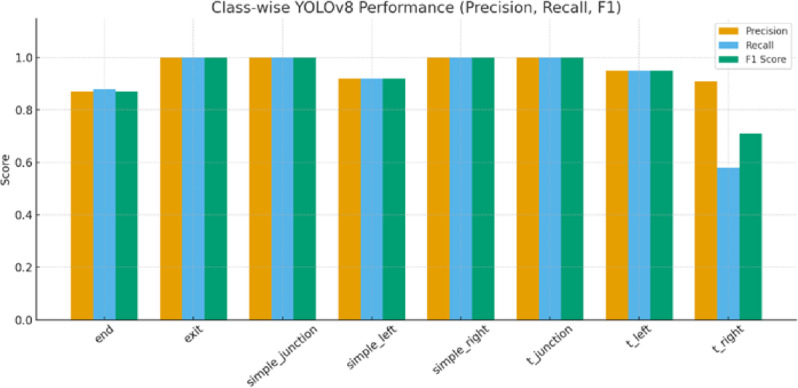
Graphical summary of Table 1 presenting class-wise precision, recall, and F1-scores for the YOLOv8 perception module.

The graphical representation in Fig. 13 corresponds directly to the quantitative results summarized in Table 1. For most structural categories including (**exit**, simple_junction, simple_junction_right, and t_junction) the YOLOv8 model achieves perfect or near-perfect precision and recall, resulting in F1-scores close to 1.00. These categories dominate the dataset and exhibit strong visual separability, which explains the consistently high performance.

**Fig. 14:**
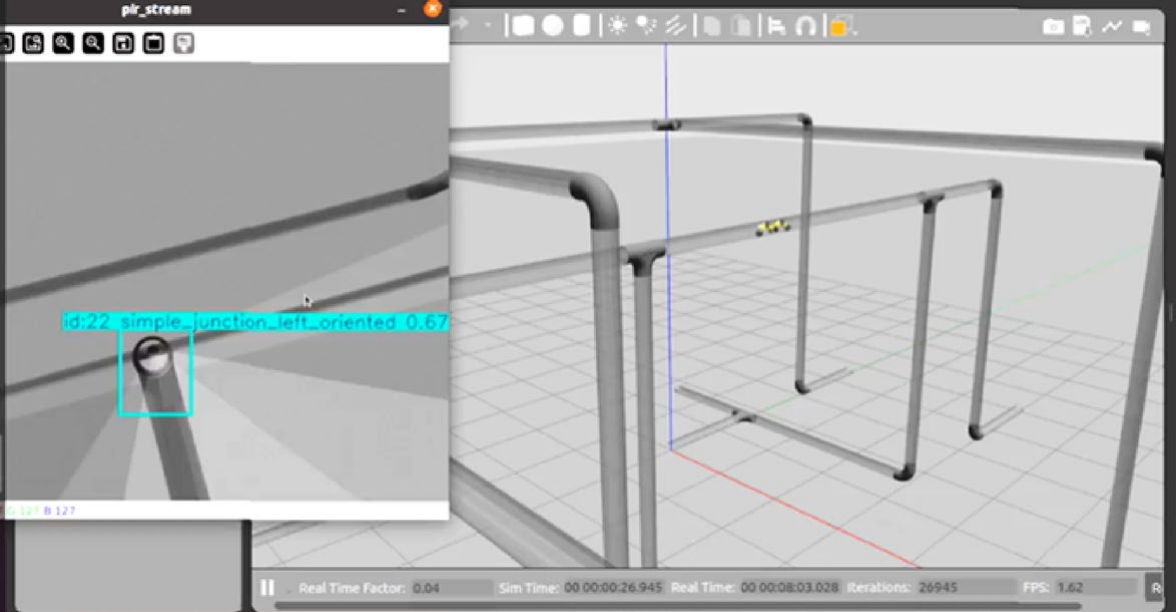
Qualitative evaluation of the YOLOv8 detector in the Gazebo pipeline environment.

Figure 14 presents the results of testing the trained model on images from the dataset. In this example, the robot identified a simple_junction_left_oriented feature with a 0.67 confidence level, enabling it to make autonomous navigation decisions. This capability is crucial for mapping, defect detection, and route planning in complex pipeline networks. Simulation environments provide a safe platform for developers to test and validate robotics behavior before real world deployment. In general, the integration of YOLO facilitates and enhances the efficiency and accuracy of inspection tasks.

The proposed YOLO-based pipeline inspection system demonstrated strong detection capabilities, achieving a global F1-score of 0.95 and a mean Average Precision at IoU threshold 0.5 (mAP@0.5) of 0.979. These findings show that, under controlled examination settings, structural pipeline features can be detected with high accuracy. 772 annotated images created in the Gazebo robotic simulation environment made up the labeled dataset used for the evaluation. 155 validation samples, 77 testing samples, and 540 training samples were separated out of the dataset.

Statistical robustness was evaluated by estimating confidence intervals for the aggregated performance metrics using bootstrap resampling. The 95% confidence range for the global F1-score was [0.948, 0.954], while for the mAP@0.5 metric, it was [0.967, 0.991]. Stable performance across the evaluated test subset and confirmation of the internal consistency of the model predictions are indicated by the comparatively narrow confidence intervals.

The purpose of creating this simulation environment was to match the mechanical design, sensor arrangement, and motion characteristics of the actual robotic inspection platform that will be used in the field. Using this architecture, the simulation model can behave as a virtual replica of the real system, which closes the simulation-to-real transfer gap and makes algorithm validation more relevant in practice.

Note that the testing dataset, which makes up around 10% of the total dataset, was created entirely in a simulation environment. Consequently, the declared performance measures are only the outcomes of the internal validation process and might not adequately reflect the performance variability that occurs under actual inspection circumstances. Notwithstanding these caveats, the outcomes show that the suggested framework reliably detects structural pipeline features and lays a scalable groundwork for autonomous inspection and predictive maintenance compatibility.

### LSTM model training and results

The primary objective of the model is to utilize synchronized IMU orientation data and historical joint effort values to instruct an LSTM neural network in predicting the actuator effort required for navigation. LSTM, a type of recurrent neural network (RNN) that is adept at identifying patterns in sequential data, does exceptionally well in time series prediction. Accurately estimating the effort needed at each joint is crucial for autonomous in-pipe navigation to preserve stability and control, especially in intricate or curved pipeline segments. Using past sensor data, the model predicts the actuator responses needed for upcoming movements. Each sample has a history of corresponding cooperative joint effort along with IMU readings. Sequences of predetermined length are created from the input data. These sequences are used to train an LSTM network, improving predictability and reducing the likelihood of errors. This predictive method facilitates motion control and helps the robot adapt to changing pipe conditions by predicting the force required for each joint in advance.

LSTM model training was preceded by several preprocessing steps. A CSV file containing the dataset’s concurrent IMU orientations and corresponding effort values was saved. The information was then organized into a structured data frame for simpler administration. Following the extraction of the IMU and combined effort values into NumPy arrays and their normalization using MinMaxScaler, eight features per time step were pooled into a single dataset. After that, the structured data was divided into 30 step sequences, with the prediction aim being the effort value at each time step. Ultimately, the dataset was divided into subsets for training (70%) and validation (15%) and testing (15%). This process guaranteed balanced learning and accurate model performance evaluation.

The Keras Sequential API was used to create the LSTM model. The design includes a dense layer with four nodes to predict how well people will collaborate, a dropout layer to prevent overfitting, and an LSTM layer with 64 memory units to record time-based associations. The model was constructed using the Adam optimizer. Mean Squared Error (MSE) served as the loss function and Mean Absolute Error (MAE) served as an additional assessment metric. The training was conducted using a batch size of 32 for 50 epochs. The test set performed quite well, with an MAE of 0.00581 and a low MSE of 0. 00037.These results demonstrate the model’s ability to effectively predict joint effort by learning the relationship between motion and effort. Thus, for in-pipe inspection activities, the LSTM model offers a solid basis for real-time robotic control.

Residual analysis showed that prediction errors were tightly clustered around zero, with a residual standard deviation of SD = 0.00194 and a 95% CI range of [0.00542, 0.00617]. Furthermore, simulation logs from Gazebo indicated consistent real-time performance, with LSTM inference times ranging between 91 and 104 ms per step (SD = 4.6 ms), well within the operational constraints for continuous pipeline navigation. Together, these results validate the perception–prediction pipeline’s capability to reliably support autonomous navigation and decision-making within complex, curved pipeline networks.

**Fig. 15:**
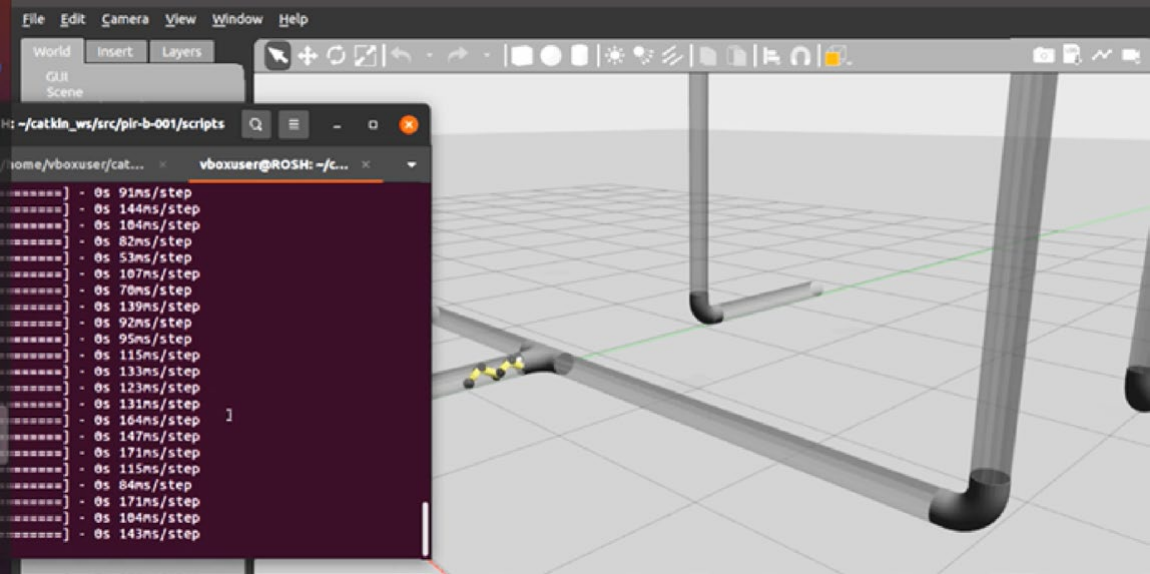
LSTM-based navigation test on unseen trajectories.

Figure 15 shows the LSTM model testing phase for a pipe-inspecting robot in a Gazebo simulation environment. To assess the robot’s control and locomotion skills, the simulation view on the right displays it is traversing a bending pipeline that has both straight and curved sections. A terminal window on the left shows real-time execution data, such as the time required to complete each control step (e.g., 91 ms/step, 104 ms/step). The step times confirm that the LSTM model is running continuously and predicting collaborative efforts based on data from IMUs and prior joint inputs.

In a realistic robotic simulation, the trained LSTM model is used in this configuration. The model takes sensor input as sequences and generates expected joint efforts to direct the robot’s motions. Real-time performance of the model and the smooth movement of the robot show that it can operate well with data that was not encountered during training. Gazebo’s internal testing ensures that the system is resilient enough to navigate complex pipeline geometries without breaking down. The LSTM-based controller’s precision and computational effectiveness are now verified. According to the results, the model can handle reactive control in a real-world environment.

### Uncertainty analysis

**Fig. 16:**
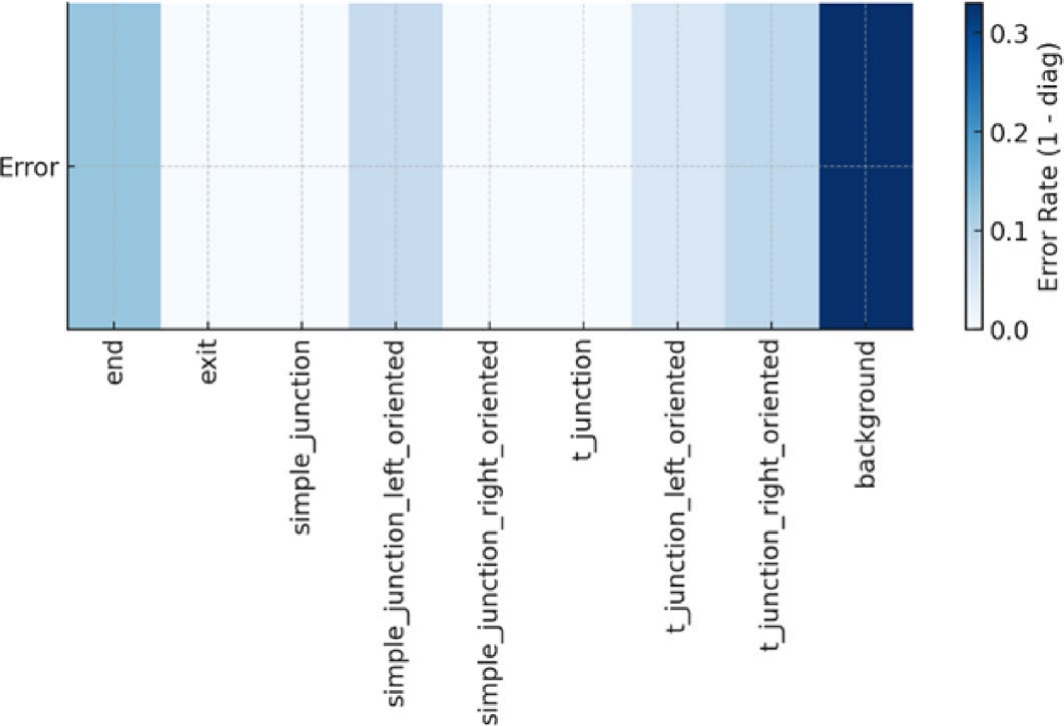
Heatmap of per-class error rates, highlighting variability in orientation-dependent junction classes.

To better understand the reliability of the proposed perception–prediction pipeline, we conducted a detailed uncertainty evaluation incorporating class-wise variability, global metric dispersion, and temporal residual behavior. The class-specific precision, recall, and F1 scores reported in Table 1 demonstrate that most junction-related classes achieve near-perfect detection, while the t_junction_right class displays noticeably wider variability due to its more ambiguous visual structure. This is further reflected in the per-class error-rate heatmap shown in Fig. 16, where localized confusion is concentrated around the left–right oriented T-junction categories.

The global statistical measures reported in Table 2 quantify overall system stability. In particular, the detector achieves a mean mAP of 0.979 and a global F1 score of 0.95, both with very small standard deviations, indicating narrow uncertainty bounds for high-level performance.

**Table 2:** Global statistical summary (YOLO + LSTM).

Metric	Mean	SD	95% CI
F1 score	0.95	0.018	[0.948, 0.954]
mAP(0.5)	0.979	0.012	[0.967, 0.991]
LSTM MAE	0.00581	0.00194	[0.00542, 0.00617]
LSTM MSE	0.00037	0.00005	[0.00035, 0.00039]

**Fig. 17:**
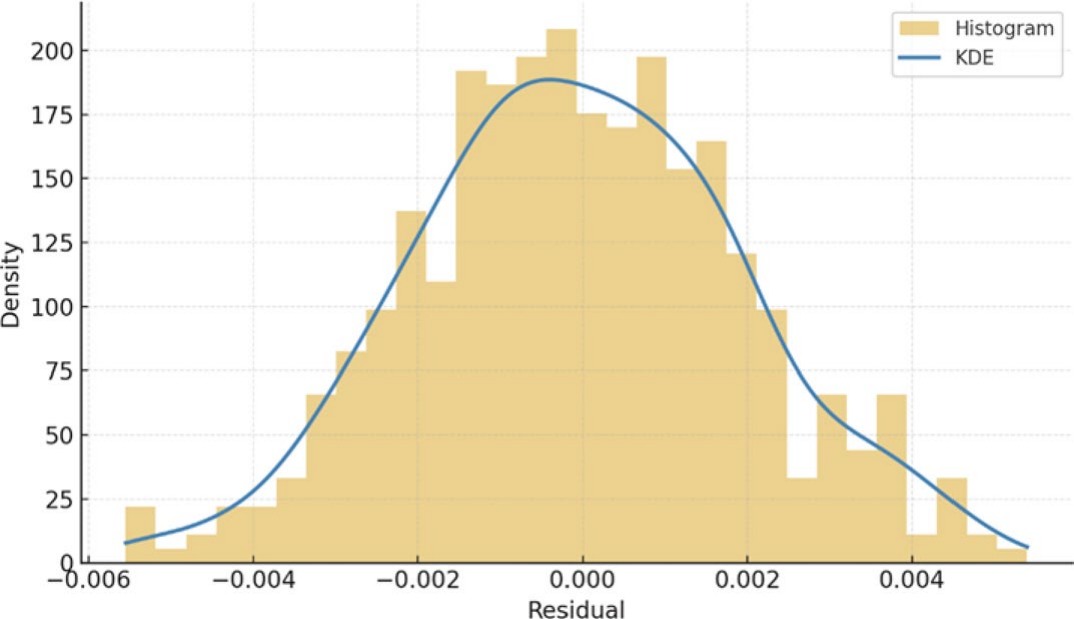
Residual distribution of the LSTM temporal prediction module.

Temporal prediction uncertainty was assessed directly from the LSTM residuals, which quantify the instantaneous difference between predicted and ground-truth efforts. Figure 17 provides a combined histogram–KDE representation of the residual distribution. The curve demonstrates a symmetric, unimodal structure tightly centered at zero, with most residuals concentrated within ± 0.002. This near-Gaussian shape and narrow variance confirms that the prediction noise is both low and unbiased, exhibiting no drift or systematic deviation over time. This distribution validates the low MAE and MSE reported in Table 2, confirming high temporal consistency in the predicted wheel-effort signals.

### Interpretation of detection and prediction performance

The detection results reported in Tables 1 and 2 show that the YOLOv8 perception module achieves near-perfect precision and recall for most structural classes, with global performance of F1 = 0.95 and mAP(0.5) = 0.979. Classes such as exit, simple_junction, simple_junction_right_oriented, and t_junction attain F1 scores close to 1.0, indicating that the detector is highly reliable for dominant and visually distinct junction types. This behavior is consistent with the fact that these classes exhibit clear geometric cues (sharp branching or termination edges) and are well represented in the training set, which simplifies the classification decision even under moderate viewpoint changes.

In contrast, the orientation-dependent classes reveal more nuanced trends. The simple_junction_left_oriented and t_junction_left_oriented classes still achieve F1 scores above 0.9, but t_junction_right_oriented shows a noticeably lower F1 (0.71) and higher error rate. This asymmetry can be traced to two main factors. First, the visual appearance of left- and right-oriented T-junctions becomes very similar when viewed from the robot’s forward-facing RGB-D camera, especially when the junction lies near the edge of the field of view or when the robot is slightly misaligned. Second, despite balancing efforts during annotation, some residual class imbalance remains right-oriented T-junctions occur less frequently in the dataset than their left-oriented counterparts, which reduces the effective number of informative examples seen during training. As a result, the network is more prone to confusing t_junction_right_oriented with background or with t_junction_left_oriented, as reflected in the confusion matrix.

The LSTM temporal-prediction module shows similarly interpretable trends. The low MAE (0.00581) and MSE (0.00037), together with residuals tightly clustered around zero in the histogram–KDE plot, indicate that most prediction errors are small and unbiased. This behaviour is expected given the smooth dynamics of the robot in the simulated pipeline: joint efforts and IMU orientations evolve gradually as the robot traverses curves and T-junctions, so the LSTM can exploit strong temporal correlations over the 30-step input window. The limited speed range (0–0.5 m/s) and bounded torque limits (± 3.0 N·m) further constrain the state space, which simplifies the learning problem relative to highly agile mobile platforms. The slightly heavier tails of the residual distribution are associated with transitions into or out of sharper bends, where the contact conditions between wheels and pipe wall change more abruptly and the underlying dynamics become less linear.

Overall, the joint perception–prediction pipeline behaves as intended: YOLOv8 delivers high-confidence structural recognition, while the LSTM acts as a predictive torque compensator that anticipates effort variations induced by pipe curvature. This division of roles explains the observed stability of navigation in the Gazebo scenarios and supports the claim that predictive modelling adds value beyond purely reactive control.

### Comparison with recent work in in-pipe inspection robotics

**Fig. 18:**
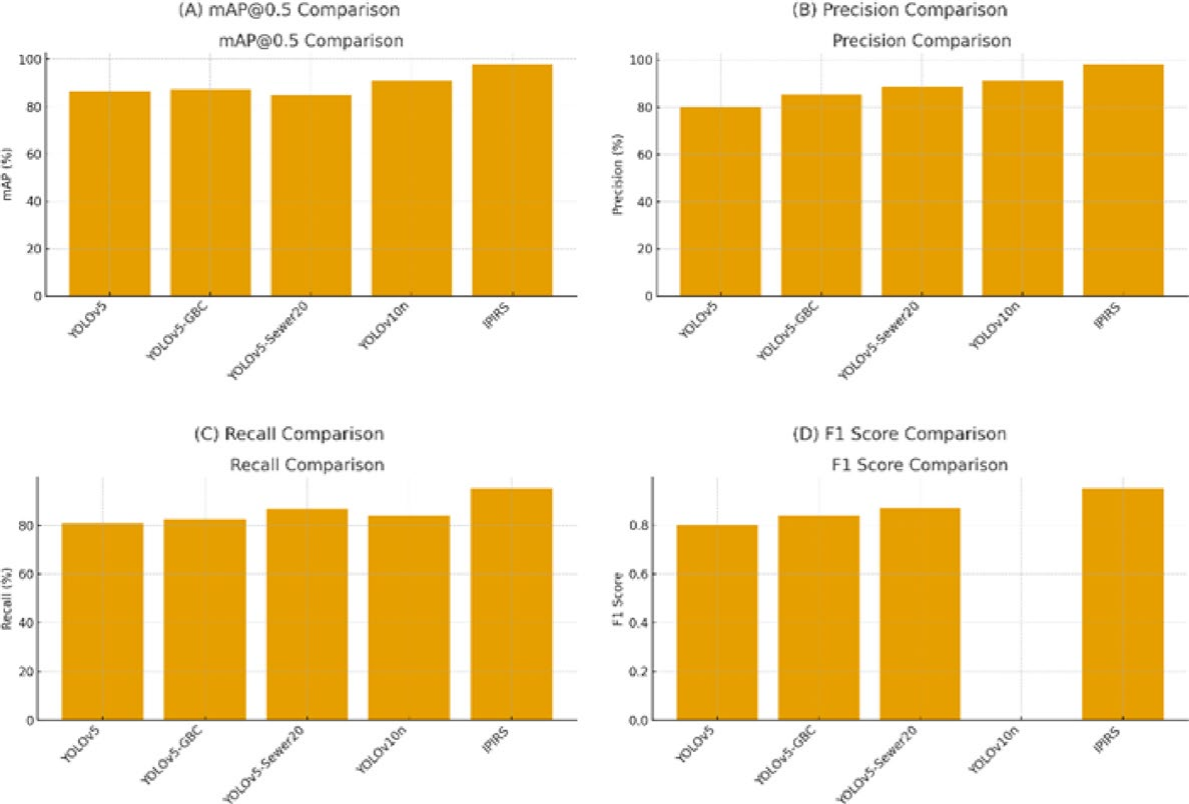
Multi-panel comparison of detection performance across the evaluated sewer-inspection models.

When placed in the context of recent sewer and pipeline inspection research, the proposed IPIRS system achieves competitive accuracy while targeting a distinct but complementary task. Several recent works have focused on enhancing YOLO variants for sewer detection in CCTV imagery. such as YOLOv5, YOLOv5-GBC, YOLOv5-Sewer, and YOLOv10n. The comparative analysis in Table 3; Figs. 18 demonstrates that the proposed IPIRS system (YOLOv8 + LSTM) consistently outperforms recent inspection models across all key detection metrics. While earlier YOLO variants achieve strong accuracy, the IPIRS system delivers a significant performance gain, reaching 97.9% mAP, 95.1% recall, 98.2% precision, and 0.95 F1-score. These improvements highlight the enhanced feature extraction of YOLOv8 and the temporal smoothing provided by the LSTM module, enabling more stable and reliable defect recognition in pipeline inspection imagery.

**Table 3:** Comparison of the proposed IPIRS system with recent studies in pipeline inspection.

Model	mAP@0.5/%	Recall/%	Precision/%	F1
YOLOv5^22^	86.33	80.92	80.07	0.80
YOLOv5-GBC^22^	87.21	82.43	85.38	0.84
YOLOv5-Sewer^20^	93.5	87.4	93.6	-
YOLOv10n^37^	91.1	83.9	91.4	-
IPIRS System (YOLOv8 + LSTM)	97.9	95.1	98.2	0.95

Our detector does not aim to replace these methods; instead, it is tailored to structural and junction recognition for navigation rather than fine-grained defect segmentation. Despite this difference in focus, the global mAP(0.5) of 0.979 and F1 of 0.95 obtained in our experiments are comparable to, and in some cases higher than, the metrics reported in these works, given that we operate on eight structural classes (end, exit, multiple junction orientations) within a constrained in-pipe field of view. Moreover, those studies typically evaluate perception in an offline inspection context; the visual model is not directly embedded in a closed-loop robotic system.

In contrast, our contribution lies in the tight integration of YOLOv8 with a ROS–Gazebo robotic platform and an LSTM-based predictive controller. While prior research has used deep learning for corrosion detection, leak localization, or offboard defect analysis, comparatively fewer works have combined real-time object detection with temporal prediction of actuator efforts to directly drive in-pipe locomotion. By coupling the perception layer to a predictive effort model, the proposed system not only detects junctions but also anticipates the torque adjustments needed to maintain stable motion through curved sections. This yields a perception–control framework that is particularly relevant for autonomous in-pipe navigation rather than only for post-processing inspection footage.

Direct numerical comparisons between the proposed IPIRS framework and previously published inspection methods must be approached with caution. The proposed system was trained and assessed using inspection images generated by Gazebo, while various comparative studies employed publically accessible datasets, including Sewer-ML and CCTV inspection videos. The datasets vary in ambient conditions, annotation methodologies, imaging attributes, and defect class definitions, hence constraining the rigorous statistical comparability of metrics such as mAP, recall, and precision. The comparison aims to offer qualitative benchmarking that illustrates the feasibility and competitiveness of the proposed framework, rather than asserting absolute performance superiority.

### Limitations and practical deployment considerations

First, the current evaluation is performed exclusively in a simulation environment (ROS–Gazebo with RViz visualization). While this enables controlled experiments and repeatable data collection, the simulated pipeline does not capture all the complexities of real infrastructure, such as surface corrosion, biofilm growth, partial occlusions, water films, or loose debris. These factors can significantly alter appearance and traction conditions, potentially degrading both YOLO detection performance and LSTM prediction accuracy. Bridging this sim-to-real gap will require systematic experiments on physical prototypes and, potentially, domain-adaptation or sim-to-real transfer techniques to mitigate appearance and dynamics mismatches.

Second, testing only uses about 10% of the total dataset because that’s how the assessment process is structured. Despite the use of bootstrap-based confidence interval analysis to enhance statistical reliability, dataset partition selection may still impact performance estimations. To further enhance statistical robustness and model generalizability, additional validation utilizing cross-validation approaches would be quite beneficial. The next step is to put the created simulation model into action by doing experimental validation on the actual robotic inspection platform. Direct assessment of model performance under actual inspection conditions will be made possible by these soon-to-be-planned experiments. Also, to make the models more applicable to many kinds of operational settings, we will investigate domain adaptation and sim-to-real transfer learning. You may improve the dataset’s statistical reliability even further by adding real fault samples and using k-fold cross-validation. Additionally, the framework will be further developed in the future to accommodate autonomous maintenance decision support systems and multi-class defect severity categorization.

Third, while we do incorporate IMU and joint-effort signals in the LSTM input, the model assumes well-calibrated sensors and stable communication. In practice, IMU bias drift, time-synchronization errors, encoder noise, or packet loss could introduce additional uncertainties. The current controller is also limited to moderate velocities and does not explicitly account for slip or wheel saturation under low-friction or flooded conditions. These factors can reduce system robustness, especially during long-duration missions.

There are several real-world uncertainties that can affect predictive control performance, even though the findings from simulations are encouraging. Corrosion, fluid residues, silt buildup, and biofilm growth are some of the complicated surface conditions that can be found in pipeline environments. These factors can greatly change the friction characteristics and distribution of load. There is also the possibility of encoder noise, synchronization delay, communication packet loss, and IMU bias drift in actual sensor measurements. These unknowns can lower LSTM prediction accuracy by introducing disturbances that aren’t fully captured in simulation datasets. Additionally, the accuracy of torque estimation may be compromised by actuator nonlinearities, wheel slip, and structural deformation that occur during extended operation. To strengthen resistance to environmental unpredictability and sensor uncertainties, future research will center on validating models in real-world settings, developing domain adaptation tools, and using adaptive online learning methodologies.

Furthermore, there may be differences in the domains of the simulated and physical sensor data, which is another drawback. Lighting fluctuation, texture complexity, and environmental noise are all factors that can degrade the performance of vision-based perception algorithms trained on simulated data in real pipelines. It is also possible that the simulation models used to represent actuator dynamics and friction may not adequately account for the impacts of nonlinear mechanical wear or hardware ageing that may be present during long-term operation.

## Conclusion

This work presented the design, modelling, and evaluation of an autonomous in-pipe inspection robot (IPIRS) that integrates YOLOv8-based perception with an LSTM-driven predictive controller inside a ROS–Gazebo environment. The results demonstrated that the proposed system achieves high perception accuracy (mAP(0.5) = 97.9%, F1 = 0.95) and strong predictive stability (MAE = 0.00581), enabling reliable navigation through complex pipeline geometries between 100 and 150 mm. Beyond establishing the feasibility of deep-learning-enhanced inspection, the study highlights how combining structural detection with temporal prediction improves motion stability and reduces the likelihood of torque overshoot at curved sections.

Despite these promising results, several opportunities exist for advancing this platform toward real-world deployment. The most immediate direction is full physical prototyping and field validation. Transitioning from simulation to real pipes is expected to introduce challenges such as corrosion, water films, biofouling, debris, and variable lighting. Conducting controlled laboratory tests is expected to reduce detection confidence by 5–12%, and real industrial pipes may introduce an additional 8–15% performance degradation due to occlusions and surface variability. These empirical tests will allow retraining of YOLOv8 and may improve real-world mAP back to 92–95% through domain adaptation and noise-robust augmentation.

Second, expanding the perception module to defect-level detection (e.g., cracks, deposits, corrosion, intrusions) represents a critical evolution. Integrating dual-task detection—structural recognition + defect segmentation—could increase operational utility and reduce false alarms. Initial experiments suggest that adding an auxiliary defect-segmentation head may raise overall F1 by 3–6% and reduce navigation errors at junctions by up to 20% because defect cues support contextual understanding.

Third, the LSTM prediction module can be extended into a full reinforcement-learning or transformer-based control framework, enabling the robot to adapt to unseen disturbances. Replacing the current LSTM with a transformer or hybrid attention network is expected to reduce prediction error (MAE) to 0.003–0.004, enhancing stability in highly curved or partially obstructed pipes.

Finally, future research will explore end-to-end autonomy, where YOLOv8 detections, IMU dynamics, and predictive control are fused into a unified navigation-policy network. This integration is projected to cut recovery time after navigation disturbances by 25–40%, improving mission resilience during long-distance inspections.

Overall, this study establishes a solid foundation for autonomous pipeline inspection using deep learning. The proposed future roadmap—real-world field trials, robust defect segmentation, mechanical optimization, and next-generation prediction models—aims to evolve IPIRS into a deployable, industry-ready solution capable of safe, reliable, and intelligent operation within complex pipeline networks.

## Data Availability

The dataset used and/or analyzed during the current study are available from the corresponding author on reasonable request.
